# Robotic single-site versus multi-port myomectomy: a case–control study

**DOI:** 10.1186/s12893-021-01245-9

**Published:** 2021-05-27

**Authors:** So Hyun Ahn, Joo Hyun Park, Hye Rim Kim, SiHyun Cho, Myeongjee Lee, Seok Kyo Seo, Young Sik Choi, Byung Seok Lee

**Affiliations:** 1 Department of Obstetrics and Gynecology, Severance Hospital, Yonsei University College of Medicine, Seoul, South Korea; 2 Institute of Women’s Life Medical Science, Yonsei University College of Medicine, Seoul, South Korea; 3 Department of Obstetrics and Gynecology, Yongin Severance Hospital, Yonsei University College of Medicine, Yongin, South Korea; 4 Biostatistics Collaboration Unit, Department of Biomedical Systems Informatics, Yonsei University College of Medicine, Seoul, South Korea; 5 Department of Obstetrics and Gynecology, Gangnam Severance Hospital, Yonsei University College of Medicine, Seoul, South Korea

**Keywords:** Robot myomectomy, Minimally invasive surgery, Robotic single-site myomectomy, Robotic multi-port myomectomy, Comparative study

## Abstract

**Background:**

This study aimed to evaluate the compatibility of robotic single-site (RSS) myomectomy in comparison with the conventional robotic multi-port (RMP) myomectomy to achieve successful surgical outcomes with reliability and reproducibility.

**Methods:**

This retrospective case–control study was performed on 236 robotic myomectomies at a university medical center. After 1:1 propensity score matching for the total myoma number, total myoma diameter, and patient age, 90 patients in each group (RSS: n = 90; RMP: n = 90) were evaluated. Patient demographics, preoperative parameters, intraoperative characteristics, and postoperative outcome measures were analyzed.

**Results:**

The body mass index, parity, preoperative hemoglobin levels, mean maximal myoma diameter, and anatomical type of myoma showed no mean differences between RSS and RMP myomectomies. The RSS group was younger, had lesser number of myomas removed, and had a smaller sum of the maximal diameter of total myomas removed than the RMP group. After propensity score matching, the total operative time (RSS: 150.9 ± 57.1 min vs. RMP: 170 ± 74.5 min, p = 0.0296) was significantly shorter in the RSS group. The RSS group tended to have a longer docking time (RSS: 9.8 ± 6.5 min vs. RMP: 8 ± 6.2 min, p = 0.0527), shorter console time (RSS: 111.1 ± 52.3 min vs. RMP: 125.8 ± 65.1 min, p = 0.0665), and shorter time required for in-bag morcellation (RSS: 30.1 ± 17.2 min vs. RMP: 36.2 ± 25.7 min, p = 0.0684). The visual analog scale pain score 1 day postoperatively was significantly lower in the RSS group (RSS: 2.4 ± 0.8 days vs. RMP: 2.7 ± 0.8 days, p = 0.0149), with similar consumption of analgesic drugs. The rate of transfusion, estimated blood loss during the operation, and length of hospital stay were not different between the two modalities. No other noticeable complications were observed in either group.

**Conclusions:**

Da Vinci RSS myomectomy is a compatible option with regard to reproducibility and safety, without significantly compromising the number and sum of the maximal diameter of myomas removed. The advantage of shorter total operative time and less pain with the same amount of analgesic drugs in RSS myomectomy will contribute to improving patient satisfaction.

## Background

Uterine myomas are diagnosed in approximately 25–40% of women during the reproductive age, causing menorrhagia, dysmenorrhea, anemia, pressure symptoms due to mass effect, and possible pregnancy-related complications [1, 2]. When conservative, symptomatic treatment for myomas fails in those wishing to preserve fertility, a laparotomy, laparoscopy, or robot-guided myomectomy is the preferred choice. Myomectomy using minimally invasive surgery (MIS) techniques has important advantages with regard to postoperative morbidity and the speed of recovery, with less intraoperative blood loss and shorter hospital stays [3, 4]. However, the limitations of ergonomics, longer learning curves, and uterine dehiscence after pregnancy due to suboptimal suture conditions are the main concerns regarding laparoscopic myomectomy, especially for deep intramural myomas [5]. When the lowest number of ports is desired, single-incision laparoscopy-guided myomectomy could also be performed; however, the added limitations of the degree of freedom for surgical tool manipulation have hindered its widespread application [6].

With the introduction of robot-guided surgery, which provides stereoscopic binocular vision, absorption of physiologic hand tremors, and wristed motion of surgical tools, minimally invasive gynecologic surgery has become more sophisticated. Robot-guided myomectomy has been accepted as a reproducible, safe, and successful means of resecting myomas without compromising the quality of the operative procedure, with delicate tools available for adequate myometrial sutures [7, 8]. However, the demand for using the lowest number of ports to induce compatible surgical outcomes has always been sought out in gynecologic surgery due to poorer cosmesis, higher costs, and the complexity of robotic multi-port (RMP) surgery compared to traditional single-port incision laparoscopic surgery [9, 10].

With the availability of robotic single-site (RSS) surgery using the Da Vinci Si system, hysterectomies and adnexal surgeries are the ones that have mainly been performed; however, myomectomies performed using the single-site system are also emerging procedures [11, 12]. Nevertheless, there is a lack of research and more challenges to overcome pertaining to RSS compared to RMP surgery, which is still the most commonly used robot-assisted approach [13]. With the initial set of non-wristed tools, the applicability of the RSS system for a wide range of gynecologic procedures had been questioned in preliminary reports [14]. However, with the addition of single-site wristed needle drivers, limitations in tissue handling and suture placement have been significantly overcome, where the 45° angulation with rotation provides better precision for driving the needle with more ease. When the characteristics of the single-site tools are properly managed, maintaining optimal triangulation between the robotic arms and the accessory port becomes possible with minimal collision of the tools, as frequently experienced with single-incision laparoscopy.

Single-site myomectomy provides additional benefits regarding the concerns over power-morcellated myoma extraction that began in 2013 [15–18]. The relative ease of in-bag manual morcellation of myomas via the 2.5-cm umbilical port and not having to modify port incision are major favorable aspects of RSS myomectomy. However, the choice of tools available for single-site procedures at present is less diverse, and the time required to skillfully coordinate the robotic arms with the assistant’s accessory port is usually longer. Validating whether Da Vinci RSS myomectomy is a reproducible and reliable method with successful surgical outcomes is a subject that needs to be addressed.

By performing a comparative analysis between the Da Vinci conventional RMP and RSS myomectomies performed at a university medical center, we aim to demonstrate whether the RSS procedure is compatible with regard to the number, size, and location of the resected myomas, as well as the technical reproducibility, safety, and surgical outcomes of the procedure.

## Methods

### Patient selection and surgical team

**Fig. 1:**
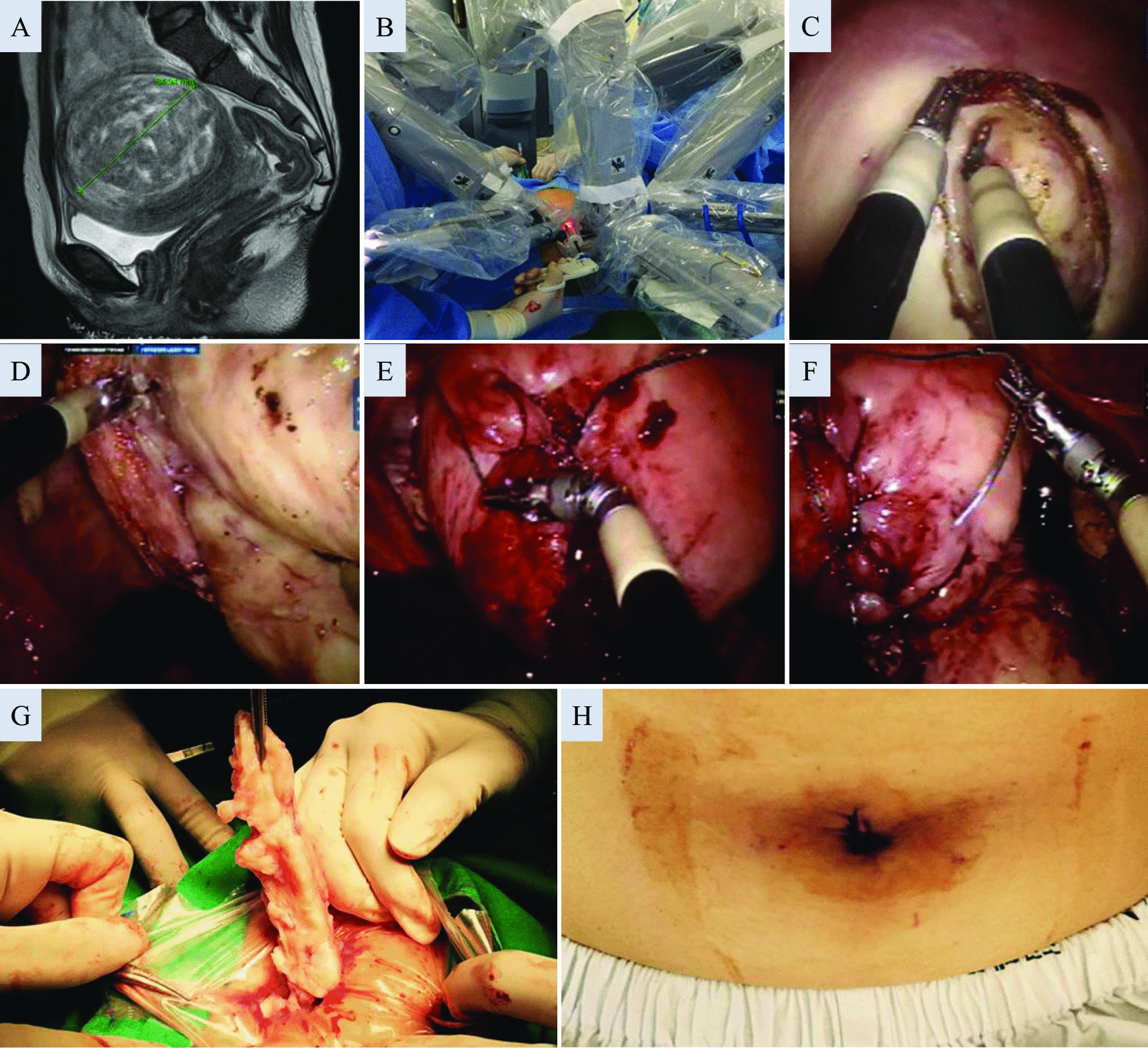
A stepwise description of a representative robotic single-site myomectomy case. Myomectomy was recommended for a 32 year-old woman due to menorrhagia and infertility for a period of 14 months. To decrease the size of this 8.5-cm FIGO type 3 myoma, leuprolide acetate was injected, but surgery could not be postponed due to intolerable heavy vaginal bleeding for 3 weeks. **A** Due to the unusual amount of bleeding and suspected distortion of the uterine cavity on ultrasonography, a pelvic magnetic resonance imaging was performed prior to the surgery (T2-weighted image). **B** A view of the operating field after docking of the Da Vinci Si single-site platform, with the first assistant to the right of the patient controlling the assistant port. The maintenance of this configuration of the robotic arms is important to create adequate triangulation intra-abdominally and to minimize tool clashing. **C** The myometrial incision was placed with a monopolar hook. **D** Although the robotic tools for the single-site platform have less variety, skillful use of bipolar forceps, the hook, and/or the wristed needle driver allows for adequate dissection. **E** Suturing was performed with V-locks in three layers, involving deep myometrial sutures to achieve adequate approximation and to prevent dead spaces from forming within the myometrium. **F** Although the serosal layer is most commonly approximated with Vicryls, when barbed suture materials are used, continuous sutures are placed so that the barbing is not exposed, and an anti-adhesive film is covered at the end. **G** Manual in-bag morcellation of the myoma with spiral incisions through the 2.5-cm umbilical port. **H** The 2.5-cm umbilical incision immediately after closure

A total of 236 patients who, in the attempt to preserve their fertility, underwent the Da Vinci Si (Intuitive Surgical, Sunnyvale, CA, USA) procedure between October 2016 and May 2021 at the Department of Obstetrics and Gynecology, Gangnam Severance Hospital, Yonsei University College of Medicine, Seoul, Korea, were included in this retrospective case–control analysis. Detailed patient characteristics including demographics, indications for surgery, intraoperative findings, and perioperative laboratory findings were reviewed and analyzed. Pelvic ultrasonography and/or magnetic resonance imaging were performed preoperatively (Fig. 1A) and 3 months postoperatively. For both types of surgeries, myomas of International Federation of Gynecology and Obstetrics (FIGO) type 2–7 were candidates for surgery, whereas patients with uteri greater in size than the size at 16 gestational weeks, those with extensive degenerative changes, or those with necrotic features present on imaging were excluded. Patients with more than 15 myomas were primarily recommended to undergo laparotomy over MIS. Patients requiring simultaneous intervention for other pelvic or abdominal conditions were excluded from the analysis. Furthermore, patients who had previously undergone laparotomies or those with suspected severe pelvic or abdominal adhesions were excluded. The surgical team was led by the same supervising surgeon who began the Da Vinci robotic surgery as a fellow in training 10 years ago, with 4 years of experience as the main operator. The supervising surgeon also had a 12-year experience in gynecologic laparoscopy.

### Operating room setup and surgical procedure

Under general endotracheal anesthesia, a patient was placed in a dorsal lithotomy and Trendelenburg position. A Rumi uterine manipulator (Cooper Surgical, Trumbull, CT, USA) was inserted into the uterine cavity.

For the single-site surgery, the standard Da Vinci Si multi-channel silicone port was used after making a 2.5-cm vertical incision on the umbilicus, with the two robotic cannulas crossing each other to create an intraperitoneal triangulation, and the 5-mm or 10-mm accessory cannulas controlled by the assistant were interchanged depending on the surgical material to be inserted. An 8.5-mm 30° high-definition camera was inserted via the camera port. Before continuing with the docking process, the pelvic and abdominal cavities were carefully inspected to check for any contraindications for the selected procedure. With the robot docked in place (Fig. 1B), the semi-rigid tools that were inserted through the curved robotic cannula to the left of the camera appeared to the right of the video screen and were operated with the surgeon’s right hand and vice versa. Such orientation of the single-site cannulas marks the key feature of a Da Vinci single-site surgery, which overcomes the limitations experienced by single-incision laparoscopic surgery. The set of tools used for the myomectomy procedure consisted of a monopolar hook, fenestrated bipolar forceps, and a wristed needle driver. Although possible, a second arm, to use the tenaculum, was not utilized for any of the cases. After the injection of diluted vasopressin when necessary, a uterine incision was made using the monopolar hook. The bipolar forceps and the monopolar hook were used to extract the myomas, while simultaneously controlling the bleeding along with the dissection process (Fig. 1C, D). When additional traction was required, a 5-mm myoma screw was controlled by the first assistant through the accessory port.

For the multi-port procedure, a 30° 12-mm camera was used, and the 8-mm trocar for the first robotic arm was placed at 8 cm left lateral and 2 cm caudal and that for the second robotic arm was placed at 8 cm right lateral and 2 cm caudal to the umbilical camera port. A 10-mm accessory port was placed in the left lower quadrant for the first assistant for manipulation. Myoma traction was achieved using the robotic tenaculum via robotic arm 1 or 2. The third arm was not used in any of the myomectomy cases.

Depending on the FIGO classification and the anatomic location of the myoma, the uterine layers were repaired in two to four layers; the myometrium, with #1-0 V-lock barbed sutures; and the serosa, with #2-0 Vicryls or #2-0 V-lock by continuous intracorporeal sutures using the conventional wristed needle driver and the single-site wristed needle driver, respectively (Fig. 1E, F). The myomas were extracted by manual knife morcellation in a polyvinyl bag via the umbilicus for the single-site procedure (Fig. 1G) and through the accessory port for the multi-port procedure, by extending the initial incision when exposure was inadequate. After thorough irrigation, either an anti-adhesive film or solution was left in the abdominal cavity, and a silastic drain was inserted through the 8-mm incision for the multi-port and at the lower tip of the umbilical incision for the single-site procedure when necessary. The umbilical incision was approximated using interrupted sutures (Fig. 1H).

Postoperative hemoglobin levels were checked routinely on postoperative day 1 and immediately when the intraoperative estimated blood loss was over 500 mL or signs of hypovolemia were present. The length of hospital stay was calculated as the period from the day of surgery to the date of discharge. The visual analog scale (VAS) for pain was assessed on days 1 and 2 after surgery. An intravenous patient-controlled analgesia (PCA) machine was used as soon as the surgery was completed. The PCA medication was prepared by an anesthesiologist with fentanyl based in a total volume of 100 mL. There was continuous background infusion throughout the postoperative period, and patients were encouraged to self-administer their own PCA medications of 1-mL bolus at a time if additional pain control was required, and self-administration was blocked for 5 min. Patients were intravenously administered pethidine at 25 mg or tramadol at 50 mg when they requested an additional analgesic. The cumulative PCA infusion volume and other analgesic medication requirements up to the second day after surgery were recorded by the medical staff. To compare the equianalgesic dose, the total fentanyl dose was calculated as the initial mixing dose of fentanyl multiplied by the total infused volume of the PCA medication, and other analgesic drug consumptions were added after conversion to fentanyl equivalents (1 mg tramadol intravenous/intramuscular is equivalent to 1 mcg fentanyl, 1 mg demerol intravenous/intramuscular is equivalent to 2.4 mcg fentanyl) [19]. All analgesic drug consumptions were summed after conversion to fentanyl equivalents. Follow-up visits were on postoperative day 7, at 3 and 9 months, and onwards, to perform imaging of the uterus and monitor any complications.

### Statistical analysis

Data were analyzed using the Kolmogorov–Smirnov test or Shapiro–Wilk test to assess normal distribution and were compared using the *t*-test for continuous variables. The Chi-square or Fisher’s exact test was used for categorical variables, as appropriate, before propensity score matching. For the analysis to be with minimized selection bias, 1:1 propensity score matching was performed for the total myoma number, total maximal diameter, and age between the RSS and RMP groups. A nearest-neighbor-matching algorithm was used along with the logit of their propensity score, with matching occurring if the difference in the logits of the propensity scores was less than 0.2 times the standard deviation of the scores (the caliper width). After propensity score matching, the paired *t*-test or McNemar’s test was used to compare the clinical parameters wherever appropriate. All statistical analyses were conducted using SAS version 9.4 (SAS Inc., Cary, NC, USA). Statistical significance was set at p < 0.05.

## Results

A total of 236 patients who underwent Da Vinci Si system-guided robotic myomectomies and had fully received routine postoperative follow-ups were analyzed. Among the 236 patients, 107 (45.3%) underwent RSS myomectomies and 129 (54.7%) received conventional RMP myomectomies. The RSS group was younger, had lesser number of myomas removed, and had a smaller sum of the maximal diameter of total myomas removed. No significant difference was observed in the proportion of obese patients with body mass index (BMI) more than 25 kg/m^2^, parity, preoperative hemoglobin levels, maximal diameter of myomas, and location of myomas according to the FIGO staging between the RSS and RMP myomectomy groups (Table 1).

**Table 1:** Patient characteristics

Parameters	DV single-site myomectomy (N = 107)	DV multi-port myomectomy (N = 129)	p-value
Age (years)	35.9 ± 6.4	37.5 ± 5.7	0.0352
BMI (kg/m^2^)			0.3458
< 25	89 (83.18)	101 (78.29)	
≥ 25	18 (16.82)	28 (21.71)	
Parity	0.3 ± 0.7	0.3 ± 0.7	0.4557
Preoperative hemoglobin level (g/dL)	12.5 ± 1.6	12.5 ± 1.7	0.9222
Number of myomas	2.5 ± 2.2	4.1 ± 3.7	0.0001
Maximal diameter of myomas (cm)	7.7 ± 2.8	7.7 ± 3	0.9217
Sum of maximal diameter of total myomas (cm)	11.3 ± 7.5	16 ± 10.3	< 0.0001
Anatomical type of myomas (%)			0.156
FIGO type 2–3	62 (57.9)	61 (47.3)	
FIGO type 4–5	18 (16.8)	34 (26.4)	
FIGO type 6–7	27 (25.2)	34 (26.4)	

p-values < 0.05 are statistically significant. Data are presented as mean ± standard deviation or n (%) values

*DV* Da Vinci, *BMI* body mass index, *FIGO* International Federation of Gynecology and Obstetrics

Since the number of myomas removed, sum of the maximal diameter of total myomas removed, and age would consequently affect surgical outcomes, we performed 1:1 propensity score matching for these three factors to minimize selection bias. After propensity matching, the number of myomas removed (RSS: 2.7 ± 2.4 vs. RMP: 2.8 ± 2.3, p = 0.4345), sum of the maximal diameter of total myomas (RSS: 11.5 ± 8 cm vs*.* RMP: 11.9 ± 7.3 cm, p = 0.683), and age (RSS: 36.1 ± 6.5 years vs*.* RMP: 37 ± 5.8, p = 0.2648) showed no significant differences between the RSS and RMP groups, with 90 patients remaining in each group (Table 2).

**Table 2:** Patient characteristics after 1:1 propensity score matching

Parameters	DV single-site myomectomy (N = 90)	DV multi-port myomectomy (N = 90)	p*-*value
Age (years)	36.1 ± 6.5	37 ± 5.8	0.2648
BMI (kg/m^2^)			0.84739
< 25	75 (83.3)	74 (82.2)	
≥ 25	15 (16.7)	16 (17.8)	
Parity	0.2 ± 0.6	0.4 ± 0.8	0.0771
Preoperative hemoglobin level (g/dL)	12.5 ± 1.6	12.5 ± 1.8	0.9861
Number of myomas	2.7 ± 2.4	2.8 ± 2.3	0.4345
Maximal diameter of myomas (cm)	7.6 ± 2.6	7.3 ± 2.9	0.683
Sum of maximal diameter of total myomas (cm)	11.5 ± 8	11.9 ± 7.3	0.6055
Anatomical type of myomas (%)			0.4132
FIGO type 2–3	52 (57.8)	41 (45.6)	
FIGO type 4–5	15 (16.7)	24 (26.7)	
FIGO type 6–7	23 (25.6)	25 (27.8)	

p-values < 0.05 are statistically significant. Data are presented as mean ± standard deviation or n (%) values

*DV* Da Vinci, *BMI* body mass index, *FIGO* International Federation of Gynecology and Obstetrics

As a result, total operative time (RSS: 150.9 ± 57.1 min vs. RMP: 170 ± 74.5 min, p = 0.0296) was significantly shorter for the single-site process. Although not statistically significant, the RSS group tended to have a longer docking time (RSS: 9.8 ± 6.5 min vs. RMP: 8 ± 6.2 min, p = 0.0527), shorter console time (RSS: 111.1 ± 52.3 min vs. RMP: 125.8 ± 65.1 min, p = 0.0665), and shorter time required for in-bag morcellation (RSS: 30.1 ± 17.2 min vs*.* RMP: 36.2 ± 25.7 min, p = 0.0684). Postoperative hemoglobin levels resulting from the loss of intraoperative blood did not result in a significant difference between the two groups. The length of hospitalization did not differ between the two surgical procedures. The VAS pain score 1 day postoperatively was significantly lower in the RSS group (RSS: 2.4 ± 0.8 days vs. RMP: 2.7 ± 0.8 days, p = 0.0149). The VAS pain scores 2 days postoperatively and the total amount of analgesic drugs used, which was calculated by converting the dose based on fentanyl, showed no differences between the two surgical procedures. There was one case of intraoperative conversion to open myomectomy in the RMP group, but it was excluded after the propensity score matching (Table 3). For 235 of the 236 cases analyzed, the pathologic reports confirmed the diagnosis of leiomyomas, except in one woman in whom a stromal tumor of unknown malignant potential (STUMP) was diagnosed, and a subsequent hysterectomy was performed 4 weeks later in this 41-year old uniparous woman. No remarkable perioperative complications other than postoperative anemia were observed in either group, and the rate of transfusion did not differ between the two modalities.

**Table 3:** Intraoperative and postoperative parameters after 1:1 propensity score matching

Parameters	DV single-site myomectomy (N = 90)	DV multi-port myomectomy (N = 90)	p-value
Docking time (min)	9.8 ± 6.5	8 ± 6.2	0.0527
Console time (min)	111.1 ± 52.3	125.8 ± 65.1	0.0665
Morcellation time (min)	30.1 ± 17.2	36.2 ± 25.7	0.0684
Total operating time (min)	150.9 ± 57.1	170 ± 74.5	0.0296
Estimated blood loss (mL)	321.3 ± 316.5	414.8 ± 415.3	0.0634
Postoperative day 1 hemoglobin (g/dL)	9.9 ± 1.5	9.7 ± 1.5	0.4593
Length of stay (days)	4.03 ± 1	4.19 ± 1.4	0.3846
Postoperative pain score on day 1 (VAS)	2.4 ± 0.8	2.7 ± 0.8	0.0149
Postoperative pain score on day 2 (VAS)	1.9 ± 0.8	2.1 ± 0.8	0.0811
Total amount of analgesic used^a^ (μg)	961.9 ± 196	988.7 ± 144.5	0.2728
Conversion to another surgical method	0	0	
Complications	20 (22.2)	29 (32.2)	0.1282
Immediate postoperative anemia	20 (100)	28 (96.5)	
Wound dehiscence	0	0	
Others	0	1 (3.4%)	
Perioperative transfusion rate (%)	14 (15.6)	14 (15.6)	> 0.9999

Data are presented as mean ± standard deviation or n (%) values

*DV* Da Vinci, *VAS* Visual analog scale

^a^For total amount of analgesics used, all analgesic drug consumptions were summed up after being converted to fentanyl equivalents

## Discussion

The combination of technique and technology is an asset of contemporary gynecologic surgery, and there is constant interest in accomplishing compatible surgical outcomes with minimal number and size of incisions as well as in finding a more ideal site of entry to the target organ. With the advent of robot-assisted surgery, the comparison among laparotomy, laparoscopic, and multi-port Da Vinci guided myomectomy has always been a subject of interest. With respect to reproducibility, safety, duration of the procedures, length of hospitalization, perioperative blood loss, likelihood of receiving transfusions, and fertility outcomes, RMP myomectomies have been reported to yield favorable outcomes, compared with laparotomy or laparoscopic myomectomies [7, 20, 21]. However, longer operative time, higher costs, and the frequent need for larger and higher numbers of ports have been discussed as unfavorable features of RMP over laparoscopic myomectomies [20–22]. With the addition of the Da Vinci single-site platform, requiring only a single umbilical incision, this system has been utilized to perform total laparoscopic hysterectomy, laparoscopic supra-cervical hysterectomy, salpingo-oophorectomy, ovarian cystectomy, and excision of endometriosis; furthermore, myomectomies using single-port systems have also been reported [12, 23, 24]. Novel methods have always been challenged over conventional techniques and have been tested over time before they are accepted as standardized methods. In the initial series of RSS surgeries, the procedures were performed using needle drivers in the absence of wrist motion, limiting their indications. However, with the subsequent introduction of wristed needle drivers as well as bipolar energy devices, more versatility has been added to the process with improved freedom of motion; therefore, the value of the current form of RSS for a variety of gynecologic procedures should be addressed.

This retrospective case–control study of RSS versus RMP myomectomy suggests that the single-site technique offers sound results without compromising the burden of the number and diameter of myomas as well as the anatomical type of myomas to be resected. The results of the present study correspond well with those of a recent systematic review that includes four studies supporting the safety of RSS myomectomy and its equivalency with the multi-port technique in terms of the most studied outcomes [25]. Two of the four studies were comparative studies [13, 26], and one of these studies conducted propensity score matching [26]. No difference was detected by both studies in the mean total operative time (145.9% vs. 147.3% and 83.3% vs. 109.2%, respectively). In addition to prior research, in our study, the total operative time was shorter in the RSM group than in the RMP group after propensity score matching. Several factors may account for the shorter duration of surgery. Unlike laparoscopic procedures, the surgeon is at the console, away from the surgical field, and a verbal order is placed for the first assistant to change the tools via the robotic arms. Typically, four to five robotic tools, including at least bipolar forceps, monopolar scissors, a tenaculum, and a needle driver, are interchanged by the assistant, consuming a significant amount of time, which has been addressed by other authors [20]. However, for the Da Vinci Si single-site platform, this waiting time is significantly reduced and the overall course is simplified, as virtually only bipolar forceps and a monopolar hook are used without substitution and are later changed to a wristed needle driver for approximation of the myometrium. Moreover, the time required for trans-umbilical manual morcellation of the myoma was considerably shorter in the RSS group (Table 2).

Several technical tips were accumulated for performing the RSS procedures. Depending on the abdominal wall thickness, the incision has to be extended beyond 2.5 cm, as the upper and lower rims of the silicone port are larger in diameter and in an hourglass shape and an Alexis retractor is useful for compressing the abdominal wall. An overzealous attempt to keep the incision as small as possible may cause circular abrasions on the skin caused by the silicone, due to the pivoting of the robotic tools, leading to a considerable amount of discomfort afterwards. When the robotic tools require more rigidity upon dissection, the length of the robotic cannula can be adjusted during the procedure to act as a rigid sheath covering the semi-rigid tools. The wristed needle driver has a smaller range of motion, with flexion of approximately 45°; however, unlike what is expected, when proper accommodation is accomplished, very little limitation in needle driving is experienced (Fig. 1F). For cases where suture placement is more difficult, two wristed needle drivers could be used for better manipulation of both tissue and suture materials. Unlike hysterectomies or adnexal surgeries where the removal of surgical specimens can be performed either via the vagina or the consistency of the mass allows for easy extraction, myomas are more difficult to extract through a small incision. Thus, powered morcellation has been widely used to facilitate the myoma extraction process. However, with a warning released in 2014 [15–18], methods for removing myomas after a minimally invasive myomectomy have been debated [27–29]. The Da Vinci single-site system could provide exemption from this burden in specimen extraction, where manual in-bag knife morcellation through a 2.5-cm umbilical incision is relatively easy to accomplish (Fig. 1G). In certain circumstances, the semi-rigid nature of RSS tools provides several benefits for the surgical process. The tools are less traumatic upon tissue handling and consider the absence of tactile sensation for the surgeon at the console, which helps to prevent tearing or puncturing tissues during the dissection process. This characteristic is especially useful for FIGO type 2 myomas, where unwanted disruption of the endometrial cavity upon dissection with robotic tools can be minimized.

Previous studies reported significantly prolonged hospital stays in patients who underwent a single-site approach than in those who underwent multi-site ones [13, 26]. However, there were no differences in the length of hospital stay between the two groups. During a patient’s hospitalization, there was no significant difference in the amount of analgesic drugs used and the pain scores on the second day after surgery between the two groups. Interestingly, the pain score on the first day after surgery was significantly lower in the RSS group. This lower pain score may be attributed to a shorter operative time and less abdominal muscle tension-induced pain with reduced number of incision sites. Reaching a lower pain score more quickly may contribute to improving a patient’s satisfaction with surgery.

Major surgical complications were not remarkable in either group, and perioperative transfusion rates for RSS myomectomy were also similar to those for the conventional multi-port method. Complications such as hernia or wound dehiscence, reported for larger umbilical incisions, were not observed in the RSS myomectomy group. Concomitant surgeries with the single-site platform were also performed, including ovarian cyst enucleation, paratubal cystectomy, and cholecystectomy; however, these cases were not included in this analysis.

There are drawbacks to the RSS procedures that the surgeon must consider during the surgical process. The smaller angulation or the absence of wrist motion limits the approach to myomas located in certain areas, arbitrarily called the ‘blind spots’; for example, if the surgeon is right handed, myomas in the left lower segment of the uterus become difficult to tackle and a deep posterior approach is required. Although the maximal diameter of myomas removed did not differ between the two groups (Table 1), when a myoma was bulky enough such that the distance from the trocar to the target was below 8–10 cm, single-site robotic arm movements became extremely limited. Adjusting the location of the port placement cephalad is less likely to be an option for single-site surgery, as placing a 2.5-cm incision above the umbilicus is not a preferred option. Moreover, the driving force of the needle driver is weaker, causing the needle to pivot in unwanted angles depending on the myometrial thickness and consistency; thus, more trial and error are required.

Careful selection of patients in the initial cases of RSS is required for trainees who have less experience with MIS, since the ergonomics may be different and they may require a longer learning curve compared with that for the conventional multi-port robotic platform. However, for an experienced MIS surgeon, the ergonomics have overlapping features in both conventional robotic surgery and laparoscopic surgery, requiring minimal accommodation periods. Additional prospective studies with a larger number of patients across different institutions should be conducted for further validation.

## Conclusions

In conclusion, Da Vinci single-site myomectomy with the use of wristed needle drivers is a compatible option with regard to reproducibility and safety, without being significantly different in the number of myomas and the total maximal diameter of myomas resected from the RMP method. The simplified nature of the single-site platform with less tool substitution and faster myoma extraction contributes to a shorter operative time without compromising the intensive suturing process. In the RSS method, the advantage of feeling less pain with the same amount of analgesic drugs, as in the RMP method, may contribute to improving patient satisfaction.

## Data Availability

The datasets used and/or analyzed during the current study are available from the corresponding author on reasonable request.

## Abbreviations

RSS: Robotic single-site
RMP: Robotic multi-port
MIS: Minimally invasive surgery
VAS: Visual analog scale
BMI: Body mass index
PCA: Patient-controlled analgesia
